# Safety and Efficacy of the Stag Beetle Knife for the Management of Zenker’s Diverticulum: An Updated Systematic Review and Meta-Analysis

**DOI:** 10.7759/cureus.104444

**Published:** 2026-02-28

**Authors:** Mannat Kaur Bhatia, Archit Garg, Lara Calegari, Panagiotis G Doukas, Mehar Bhatia, Oghenefejiro O Ogwor, Sotirios Doukas, Babu Pappu Mohan, Arkady Broder

**Affiliations:** 1 Gastroenterology, Saint Peter's University Hospital/Rutgers Robert Wood Johnson Medical School, New Brunswick, USA; 2 Internal Medicine, Saint Peter's University Hospital/Rutgers Robert Wood Johnson Medical School, New Brunswick, USA; 3 Internal Medicine, Saint Peter’s University Hospital/Rutgers Robert Wood Johnson Medical School, New Brunswick, USA; 4 Medicine, Saint Peter's University Hospital/Rutgers Robert Wood Johnson Medical School, New Brunswick, USA; 5 General Medicine, Government Medical College Patiala, Patiala, IND; 6 Internal Medicine, Saint Joseph Hospital, Bangor, USA; 7 Gastroenterology, Orlando Gastroenterology, PA, Orlando, USA

**Keywords:** dysphagia, meta-analysis, stag beetle knife, systematic review, zenker's diverticulum

## Abstract

The stag beetle (SB) knife, a unique scissor-shaped device with rotating insulated monopolar blades, is increasingly used in the endoscopic management of Zenker's diverticulum (ZD). This systematic review and meta-analysis aimed to evaluate its overall safety, efficacy, and feasibility. A thorough search of electronic databases and conference abstracts was conducted until November 2023. Meta-analysis utilized the random-effects model, with I^2 ^(%) assessing heterogeneity. Subgroup analysis was based on sample size, employing the standard mean difference (SMD) and 95% confidence interval (CI) for continuous variables. Key outcomes included clinical success, recurrence rate, adverse events, and improvement in dysphagia score. Eight studies with 299 patients (60.5% males, mean age: 72.75±2.86 years, ZD size: 2.66±0.52 cm, and procedures lasted 23.06±10.00 min) were included. Clinical success was achieved in 86% (95% CI: 81-90; I^2^=0%) after 10.98 sessions, and 22.74% required multiple (up to four) sessions. The recurrence rate was 15% (95% CI: 11-20; I^2^=0%). Intraprocedural complications occurred in 9% (95% CI: 5-13; I^2^=29%), primarily minor bleeding (9.6%), micro-perforation (2%), odynophagia (1.3%), and fever (1.05%). Late-onset bleeding occurred in 3.2% after one week. Adverse events were conservatively managed, and subgroup analysis by sample size indicated a significant difference in mean sessions (p=0.02). Dysphagia scores showed significant improvement following treatment, with an SMD of -1.59 (95% CI: -2.27 to -0.91; p<0.01; I²=97%) over a mean follow-up of 22.23±11.47 months. The meta-analysis confirms the SB knife's success in ZD, displaying excellent safety and dysphagia improvement. However, further research is needed to define optimal patient cohorts and compare them with other management techniques.

## Introduction and background

Zenker's diverticulum (ZD), a pulsion diverticulum emerging in the weakened posterior hypopharyngeal area known as Killian's triangle, is a relatively uncommon condition, affecting 0.01-0.11% of the American population, notably prevalent among males in their seventh and eighth decades of life [1]. Despite the prevalence, the exact pathophysiology of ZD remains partially understood. The primary hypothesis suggests that motor abnormalities in the upper esophageal sphincter (UES) facilitate herniation of the esophageal mucosa through Killian's triangle [2]. ZD can either remain asymptomatic or manifest with various symptoms, such as dysphagia, regurgitation, cough, aspiration, foreign body sensation, and weight loss [1,2]. Symptom development correlates with both UES motor dysfunction and the accumulation of ingested material in the diverticular pouch, contingent on diverticulum size [1,2]. Consequently, ZD treatment aims to address motor abnormalities through myotomy of UES muscles, potentially involving suspension or resection of the diverticular pouch [3].

Treatment options for ZD encompass open surgery, rigid endoscopy, and flexible endoscopy, particularly flexible endoscopic septum division (FESD) [4]. In contrast to open surgery and rigid endoscopy, FESD offers advantages by obviating the need for general anesthesia and neck hyperextension. The procedure entails incising the mucosa and muscular fibers of the diverticular septum, achieving a partial myotomy of the cricopharyngeal muscle, and creating a passage for clearing ingested materials from the diverticular pouch [5]. Introduced in 1995, FESD has undergone various modifications and employed diverse cutting devices, signifying a substantial evolution in ZD treatment techniques [6].

Zenker's diverticulum (ZD) is a mucosal outpouching of the cervical esophagus through the Killian triangle [7]. When compared to endoscopic procedures, the surgical approach has much higher procedure-related morbidity and hospital stay. Among endoscopic methods, the flexible endoscope method is far less complicated and less invasive than the rigid endoscope method [8]. Several instances of intraluminal therapy of ZD using a flexible endoscope have been published, with various procedures available - needle-knife [2], hook-knife, monopolar forceps [9], argon plasma coagulation, and so on [10].

The stag beetle (SB) knife is a novel scissor-shaped, rotating device with two insulated monopolar blades increasingly used in the endoscopic management of Zenker's diverticulum (ZD) [11]. This study aimed to assess the pooled safety, efficacy, and feasibility of using the SB knife for the management of ZD.

This article was previously published as a preprint on the medRxiv server on April 27, 2020.

## Review

Materials and methods

Protocol and Eligibility Criteria

This systematic review, conducted per the Preferred Reporting Items for Systematic Reviews and Meta-Analyses (PRISMA) guidelines, comprehensively assessed the efficacy of the stag beetle knife in managing Zenker's diverticulum (ZD) through a meta-analysis of studies. Inclusion criteria were as follows: (1) studies evaluating SB knife application in ZD endoscopic management, (2) human subjects with symptomatic ZD, (3) minimum follow-up duration >2 months, (4) peer-reviewed publications in English, and (5) sample size ≥10 patients. Exclusion criteria were as follows: (1) comments, editorials, review articles, small case series, and case reports, (2) studies lacking outcome and/or follow-up data, (3) animal model studies, (4) previously published series with overlapping data, and (5) non-English language publications.

To ensure efficiency in data collection and avoid redundancy, cases with multiple publications prioritized data extraction from the most recent or comprehensive reports. Additionally, papers on flexible endoscopy for symptomatic ZD patients were included, requiring only the utilization of flexible endoscopic treatment and a follow-up duration exceeding two months, while exclusion criteria comprised comments or review articles, lack of outcome and/or follow-up data, exclusive use of animal models, studies with fewer than 10 patients, and the expansion of previously published series. This meticulous approach underscores the commitment to a thorough assessment of the stag beetle knife's efficacy in ZD management.

Literature Search, Study Selection, and Data Extraction

Conducting a rigorous literature review, this systematic analysis adhered to the Preferred Reporting Items for Systematic Reviews and Meta-Analyses (PRISMA) guidelines. Employing comprehensive search strategies, this meta-analysis delved into the efficacy and safety of the stag beetle knife in treating Zenker's diverticulum (ZD). Database searches, including PubMed, Cochrane, and MEDLINE, were executed from their inception to November 2023. A systematic algorithm was applied to PubMed and Embase databases. The algorithm used for PubMed was (("Zenker Diverticulum"[Mesh] OR "Zenker Diverticulum"[All Fields] OR "Zenker's Diverticulum"[All Fields] OR "Zenker's Diverticula"[All Fields] OR "Zenker Diverticula"[All Fields] OR "pharyngeal pouch"[All Fields]) AND ("endoscopy"[MeSH Terms] OR "endoscopy"[All Fields] OR "endoscopic"[All Fields] OR "flexible endoscopy"[All Fields] OR "peroral endoscopic myotomy"[All Fields] OR "FESD"[All Fields]) AND ("stag beetle knife"[All Fields] OR "SB knife"[All Fields] OR "SB Knife Jr [All Fields] OR "scissor-type knife"[All Fields]) AND English[lang]) AND (("2013"[PDAT]: "2023"[PDAT])). The algorithm used for EMBASE was ('zenker diverticulum'/exp OR 'zenker diverticulum' OR 'zenker's diverticulum' OR 'pharyngeal pouch') AND ('endoscopy'/exp OR 'endoscopy' OR 'endoscopic' OR 'flexible endoscopy' OR 'fesd') AND ('stag beetle knife' OR 'sb knife' OR 'scissor knife') AND [english]/lim AND (2013-2023)/py. The algorithm used for Cochrane Library was ("Zenker diverticulum" OR "Zenker's diverticulum" OR "pharyngeal pouch") AND ("endoscopy" OR "endoscopic" OR "FESD") AND ("stag beetle knife" OR "SB knife"). The inclusion criteria focused on studies involving human subjects, published in English-language, peer-reviewed journals, and examining the stag beetle knife's application in ZD endoscopic management, irrespective of the techniques used.

After eliminating duplicates, two independent reviewers (MKB, BPM) meticulously screened titles and abstracts, ensuring alignment with the research question and pre-specified criteria. Full-text retrieval followed, and eligibility screening was conducted, resolving discrepancies through co-author consultation. Data extraction from selected studies, performed independently by three reviewers, covered study design, patient demographics, clinical features, symptom evaluation criteria, adverse events, treatment success, follow-up duration, and recurrences. The primary clinical outcomes assessed the success rate, adverse event rate, and recurrence rate of stag beetle knife treatment in ZD. Subgroup analyses were conducted based on sample size, evaluating mean dysphagia score reduction and the number of sessions required.

Quality Assessment

Conducting a methodological quality assessment, two reviewers independently evaluated the susceptibility to bias through the methodological index for non-randomized studies (MINORS) quality score, adapting items to suit the review's scope. Resolution of any divergent quality assessments between these two contributors involved seeking guidance from co-authors. Following this, a team of at least two authors (MKB, BPM) extracted data on study-related outcomes from individual studies and entered them into a standardized form.

Statistical Analysis

Clinical data from individual studies were analyzed for summary statistics, reporting patient age as median (interquartile range {IQR}) or mean (SD) for continuous variables and gender as counts and percentages for categorical data. Meta-analysis, employing the random-effects model, assessed outcomes such as clinical success, recurrence rate, adverse events, and dysphagia score improvement. Heterogeneity was gauged through Cochran Q, I^2^ statistics, and a 95% prediction interval. Subgroup analysis, based on sample size, utilized the standard mean difference (SMD) with 95% CI for continuous variables. Meta-regression examined covariates, including sociodemographic attributes, methodological factors, and clinical/technical parameters. All analyses were conducted using the meta-package software and R programming (version 4.3.2; Vienna, Austria: R Foundation for Statistical Computing). Event rates for outcomes in each study were calculated as proportions, and the I^2^ statistic guided the use of a random-effect model in cases of substantial heterogeneity (I^2^>50%).

Results

Search Results and Population Characteristics

A total of eight studies met the inclusion criteria. They comprised 299 patients (60.5% male), with a mean age of 72.75±2.86 years and a mean ZD size of 2.66±0.52 cm. The mean procedure duration was 23.06±10.00 min.

Characteristics and Quality of Included Studies

**Figure 1:**
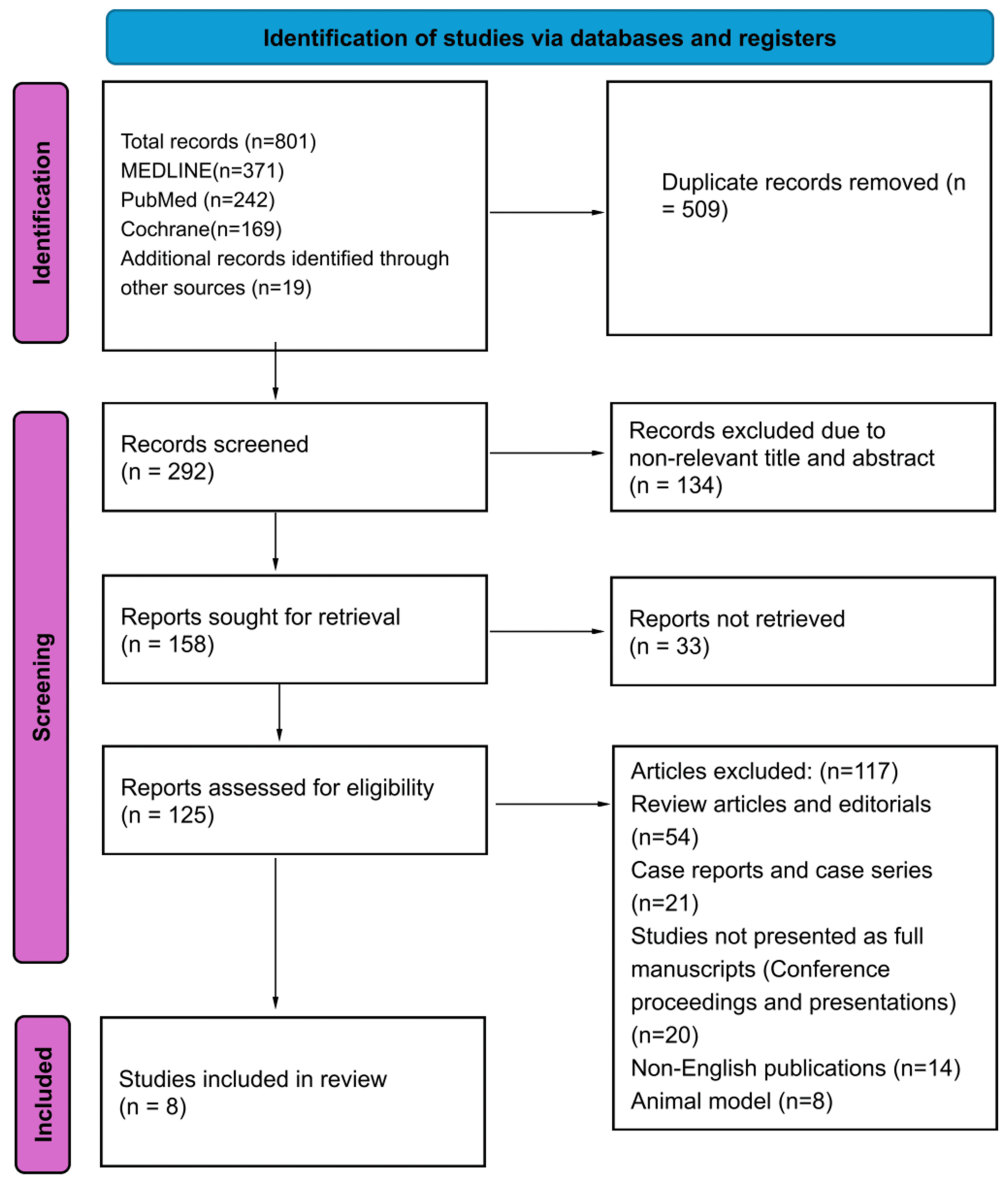
PRISMA study selection flow chart. PRISMA: Preferred Reporting Items for Systematic Reviews and Meta-Analyses

Out of 801 initially identified records, 509 duplicates were removed. Among the articles reviewed, eight studies met the inclusion criteria for the meta-analysis, encompassing various study designs, such as retrospective cohort, retrospective case-control, cross-sectional, prospective cohort, and prospective case-control (Figure 1). The distribution of study designs included four prospective, two retrospective, and two observational studies. According to the MINORS scale, all studies demonstrated moderate or high quality. The average follow-up period was 22.24 months, and the mean Zenker's diverticulum (ZD) size was 2.66 cm (Table 1). Notably, three studies were deemed high quality, with the remaining studies classified as medium quality; no low-quality studies were identified (Table 2).

**Table 1:** Study characteristics table for systematic review. DISR: double incision and snare resection; FESD: flexible endoscopic septum division; CP: cricopharyngeal; ZD: Zenker's diverticulum

Studies	Design	Participants characteristics	Follow-up (months)	ZD size (cm)	Success rate (%)	Complications (majority type)	Severe adverse events	Recurrence rate (%)	Dysphagia score (before)	Dysphagia score (after)	Technique used
Goelder et al. (2016) [12]	Prospective cohort	Total n=52, age: 72 years, sex (M/F): 34/18, BMI: 24.4	16	3	90.38	9.6% (bleeding)	None	11.53	2±0.75	1±1.0	Mucomyotomy
Manzeneder et al. (2021) [13]	Prospective cohort	Total n=100, age: 71 years, sex (M/F): 64/36, BMI: 26.1, weight loss: 2.3	41	2	83	12.7% (bleeding)	None	17	3.45	1.09 (p<0.001)	47.6% modified DISR, 52.4% single incision
Devani et al. (2020) [14]	Prospective cohort	Total n=20, age: 70 years, sex (M/F): 11/9, BMI: 23.3	27	3	90	2.5% (bleeding)	None	10	3±0.5	1±0.75	Flexible endoscopy
Ishaq et al. (2020) [15]	Retrospective observational	Total n=65, age: 74 years, sex (M/F): 26/39, BMI: 25.7	19	2.4	75.4	6.2% (bleeding, hypoxia)	None	24.6	2±0.5	1±0.5	FESD
Battaglia et al. (2015) [16]	Retrospective analysis	Total n=31, age: 71 years, sex (M/F): 25/6, BMI: 33.2	7	3	87.1	3.2% (late-onset bleeding)	3.2% (bleeding)	16.1	2±0.75	0±0.05	Flexible endoscopy
Toro-Ortiz et al. (2022) [17]	Descriptive observational	Total n=12, age: 70.5 years, sex (M/F): 8/4, BMI: 26.5	12	3.25	75	28.5% (odynophagia)	None	25	Not reported	Not reported	Endoscopic septotomy
Ramchandani and Reddy (2013) [18]	Observational human study	Total n=3, age: 73 years, sex (M/F): 3/0, BMI: 24.8	24	2.2	100	33.33% (bleeding)	None	Not reported	2±0.38	1±0.75	CP myotomy
Outomuro et al. (2020) [19]	Prospective cohort	Total n=16, age: 78 years, sex (M/F): 10/6, BMI: 31.0	23.41	2	87.5	1.3%	None	11.3	1.96±0.68	0.25±0.52	Flexible endoscopy

**Table 2:** Quality of included studies according to MINORS criteria. MINORS: methodological index for non-randomized studies

MINORS score	Goelder et al. (2016) [12]	Manzeneder et al. (2021) [13]	Devani et al. (2020) [14]	Ishaq et al. (2020) [15]	Battaglia et al. (2015) [16]	Toro-Ortiz et al. (2022) [17]	Ramchandani and Reddy (2013) [18]	Outomuro et al. (2020) [19]
Clearly stated aim	2	2	2	2	2	2	2	2
Inclusion of consecutive patients	2	2	2	2	2	2	2	2
Prospective collection of data	1	0	0	1	2	1	0	1
Endpoints appropriate for the aims of the study	1	1	0	1	2	1	0	0
Unbiased assessment of the study endpoints	0	0	1	1	2	0	1	1
Follow-up period appropriate for the aims of the study	1	2	2	2	2	2	1	1
Loss to follow-up <5%	0	1	1	1	0	2	2	1
Total	7	8	8	10	12	10	8	8

Meta-Analysis Outcomes

Clinical success was achieved after a mean of 10.98 (2.63-19.32); I^2^=93% number of sessions, and 22.74% patients required more than one treatment session (up to four) to achieve clinical remission. The pooled clinical success rate, defined as symptomatic remission after the first procedure, was 86% (95% CI: 81-90; I^2^=0%). The pooled recurrence rate, defined as relapse of symptoms after the first intervention, was 15% (11-20; I^2^=0%). The pooled prevalence of intraprocedural complications encountered during the procedure was 9% (5-13; I^2^=29%). The majority were minor bleeding (9.6%), followed by micro-perforation (2%), odynophagia (1.3%), and fever (1.05%). Only one study reported a case of late-onset bleeding in 3.2% of patients after one week. All these adverse events were managed conservatively, including endoscopic management of bleeding (Tables 3, 4).

**Table 3:** Random-effect model results of primary outcomes of stag beetle knife in ZD. ZD: Zenker's diverticulum

Stag beetle knife in ZD - primary outcomes	Pooled proportions	Number of studies
Clinical success rate	86% (95% CI: 81-90; I^2^=0%)	8
Recurrence rate	15% (95% CI: 11-20; I^2^=29%)	7
Complication rate	9% (95% CI: 5-13; I^2^=0%)	8

**Table 4:** Random-effect model results of secondary outcomes of stag beetle knife in ZD. ZD: Zenker's diverticulum

Stag beetle knife in ZD - secondary outcomes	Standard mean deviation	Number of studies	p-Value
The mean number of sessions	10.98 (95% CI: 2.63-19.32; I^2^=97%)	7	<0.01
Improvement in dysphagia score	-1.59 (95% CI: -2.27 to -0.91; I^2^=93%)	4	<0.01

**Figure 2:**
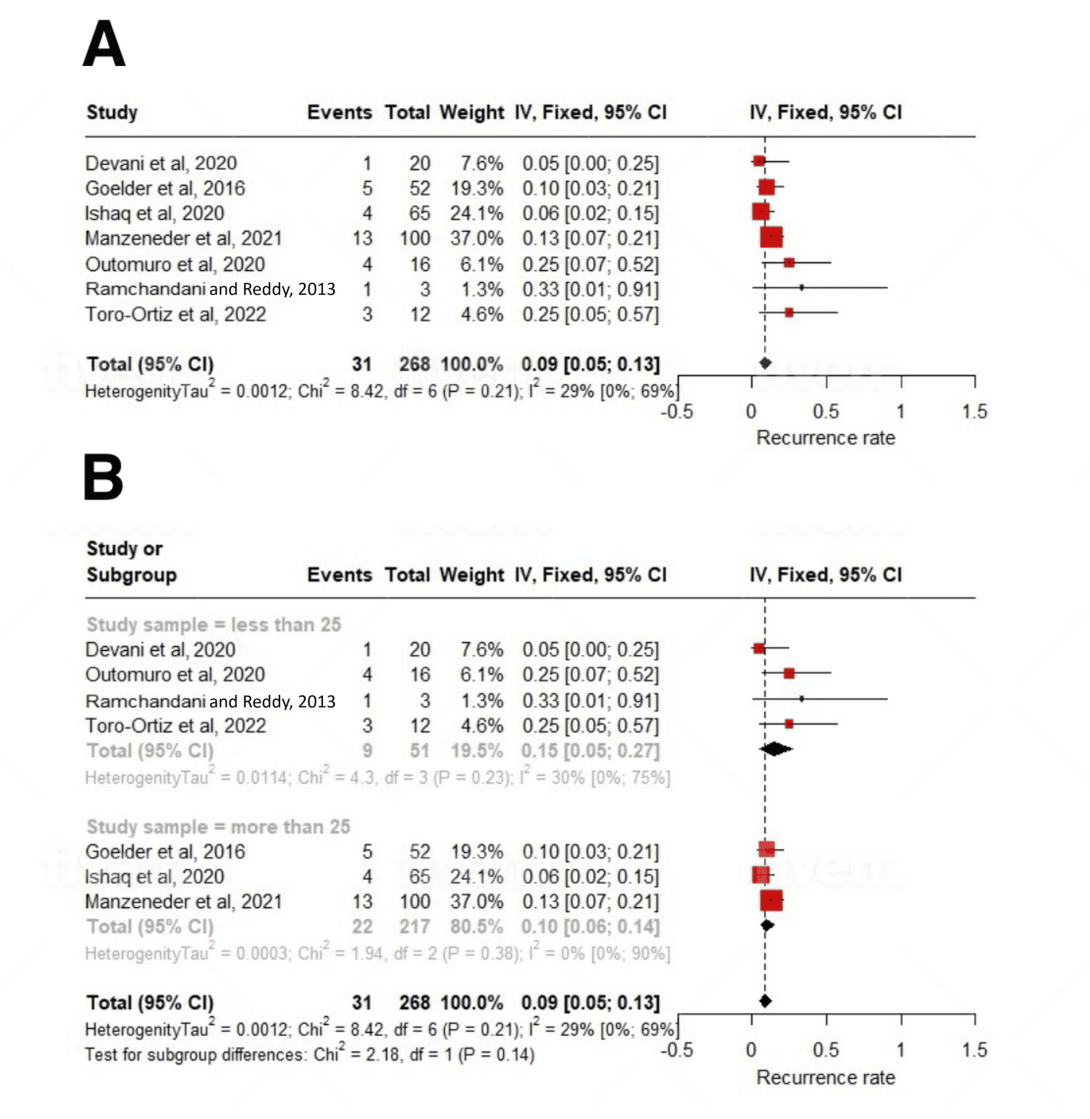
Forest plot of complication rates. (A) Overall analysis and (B) subgroup analysis [12-15,17-19].

**Figure 3:**
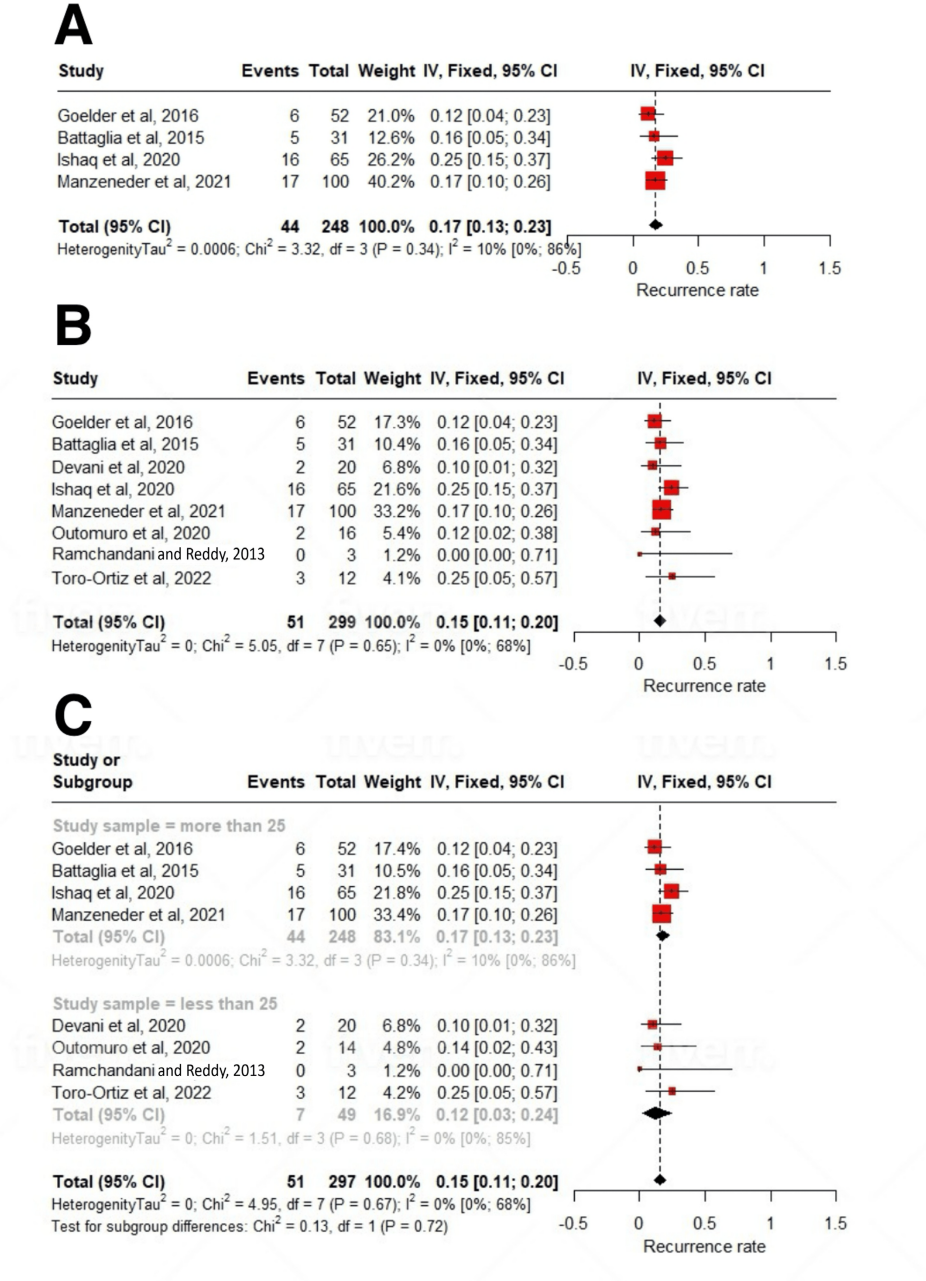
Forest plot of recurrence rates. (A) Studies with >25 participants, (B) overall analysis, and (C) subgroup analysis [12-19].

**Figure 4:**
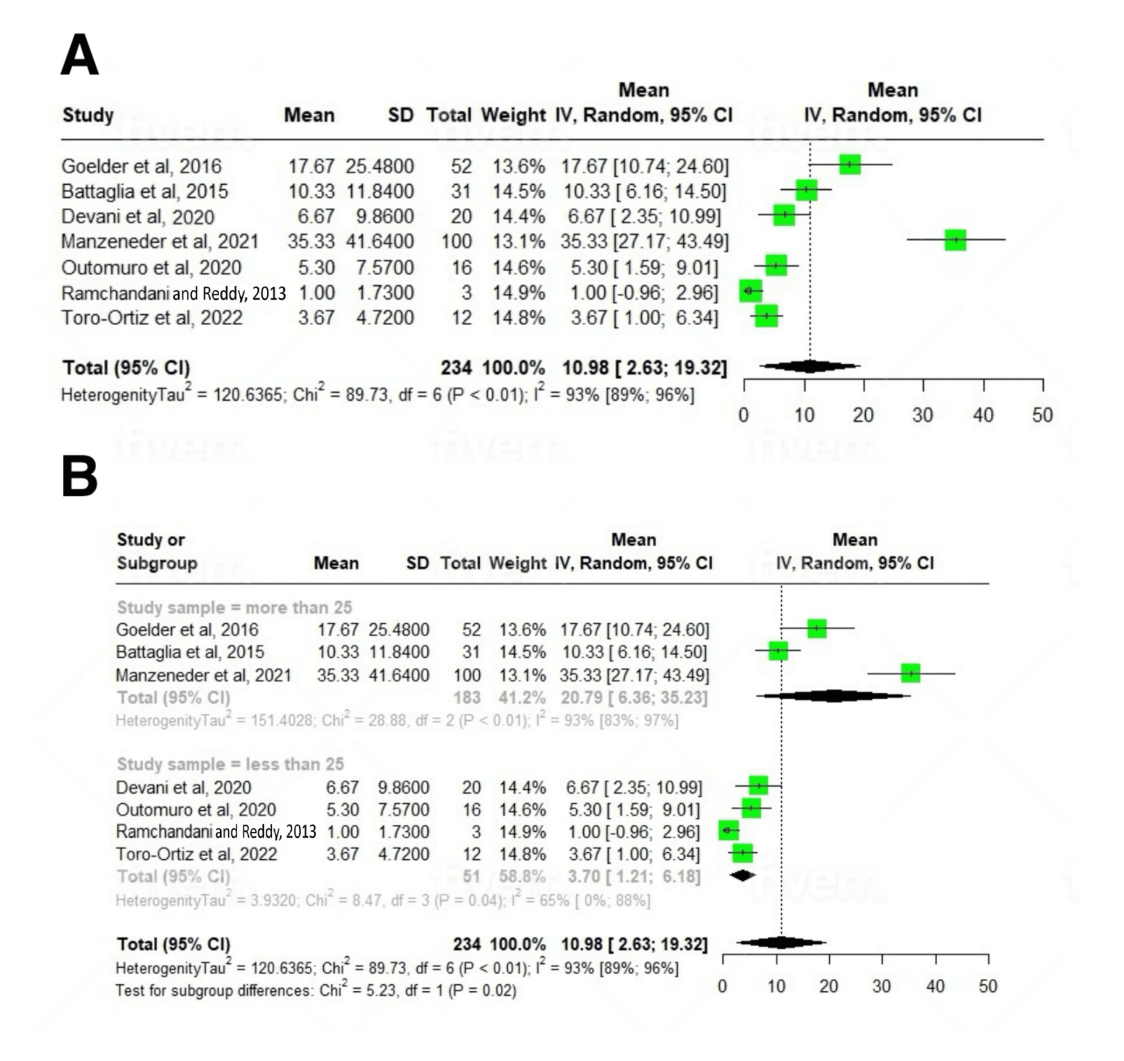
Forest plot of procedure sessions for ZD treatment. (A) Overall analysis and (B) subgroup analysis [12-14,16-19]. ZD: Zenker's diverticulum

**Figure 5:**
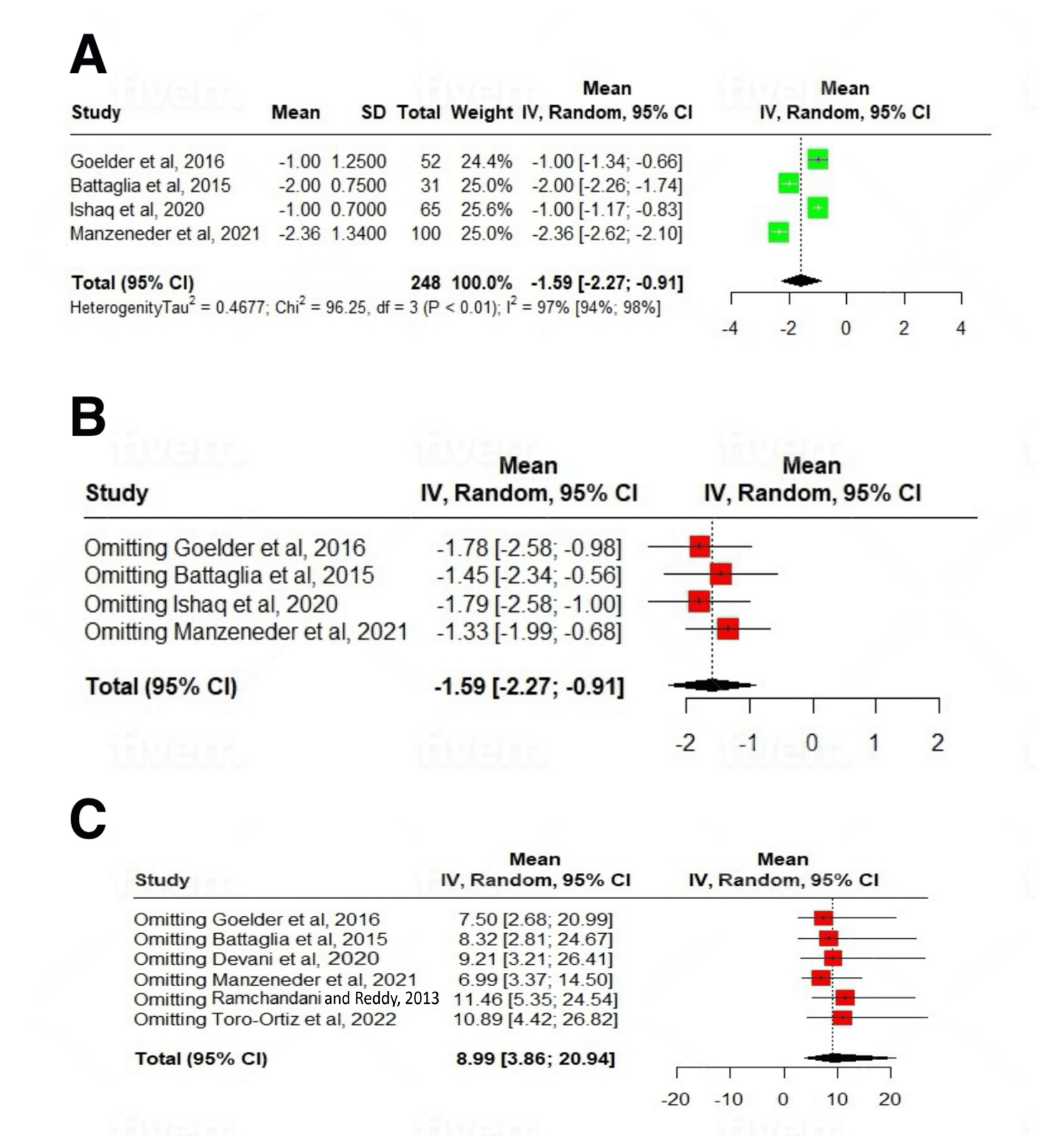
Forest plot of dysphagia scores. (A) Studies with >25 participants, (B) leave-one-out analysis, and (C) leave-one-out meta-analysis for session number [12-18].

**Figure 6:**
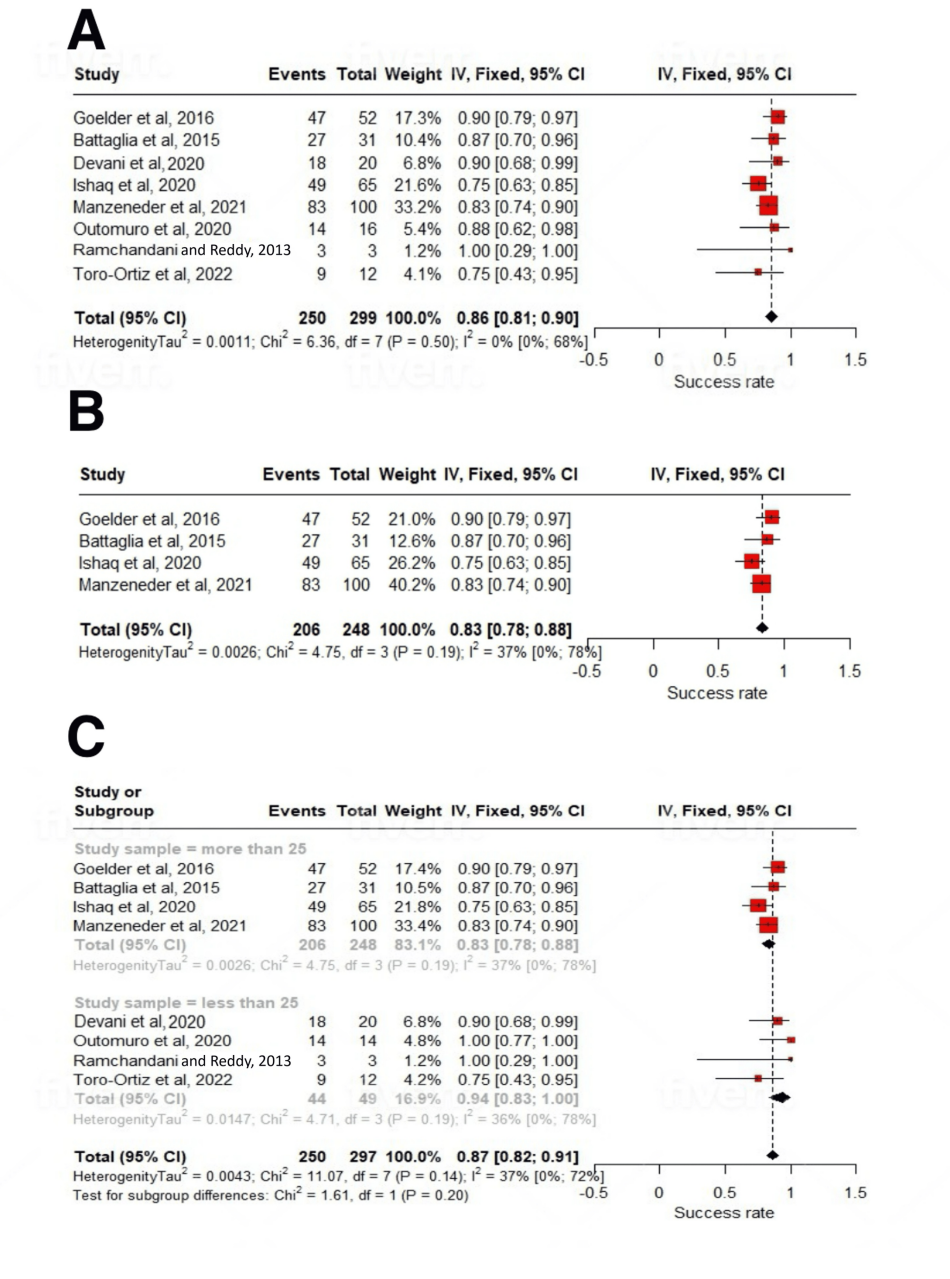
Forest plot of success rates. (A) Overall analysis, (B) studies with >25 participants, and (C) subgroup analysis [12-19].

On subgroup analysis based on study sample size, a statistically significant subgroup difference was seen (p=0.02) for the mean number of sessions required. For studies with n>25, it was 20.79 (6.36-35.23); I^2^=93%, and for studies with n<25, it was 3.7 (1.21-6.18); I^2^=65% number of sessions. After a mean follow-up period of 22.23±11.47 months, the treatment was associated with a significant reduction in dysphagia scores (SMD: -1.59; 95% CI: -2.27 to -0.91; p<0.01) (Figures 2A, 2B, 3A-3C, 4A, 4B, 5A-5C, 6A-6C).

Validation of meta-analysis results

Sensitivity Analysis

To assess whether any one study had a dominant effect on the meta-analysis, we excluded one study at a time and analyzed its effect on the main summary estimate. In this analysis, no study significantly affected the outcome or the heterogeneity.

Heterogeneity

We assessed the dispersion of the calculated rates using the prediction interval (PI) and I^2^% values. The PI gives an idea of the range of the dispersion, and I^2^ tells us what proportion of the dispersion is true rather than chance. Despite high I^2^% values for some outcomes (particularly number of sessions {I^2^=97%} and dysphagia score improvement {I^2^=93%}), the PIs provide important context. For example, while the I^2^ statistic indicates that 97% of the variability in session numbers is due to true heterogeneity rather than chance, the prediction interval (2.63-19.32) suggests that future studies would likely report mean session numbers within this range. This heterogeneity is primarily explained by differences in study sample sizes, as demonstrated by our subgroup analysis (p=0.02).

Publication Bias

Formal statistical tests for detecting publication bias, such as Egger's regression test or visual inspection of funnel plot symmetry, are considered underpowered and unreliable when the number of studies is this small. Hence, assessment of publication bias was not performed, as the meta-analysis included fewer than 10 studies.

Discussion

In our attempt to investigate the efficiency and safety of endoscopic management of ZD by SB knife, we performed a meta-analysis and systematic review of relevant literature. The meta-analysis incorporated eight studies encompassing 299 patients undergoing stag beetle knife treatment for Zenker's diverticulum (ZD). These studies reported clinical outcomes, follow-up observations, and adverse events in 299 individuals who underwent endoscopic treatment for ZD. Study designs included prospective cohorts [12-14], retrospective observational studies [15], retrospective analyses [16], descriptive observational studies [17], and observational human studies [18,19]. The predominant technique employed across studies varied, with mucomyotomy, modified double incision and snare resection (DISR), single incision, flexible endoscopy, FESD, endoscopic septotomy, and cricopharyngeal (CP) myotomy. Pooling data from 299 individuals was performed in order to compare and statistically analyze clinical outcomes of treatment with the SB knife in individuals with ZD, as well as to identify the disease recurrence rate and the degree of post-procedure complications. The pooled clinical success rate was 86%, with a mean of 10.98 sessions required. The recurrence rate stood at 15%, and intraprocedural complications were observed in 9% of cases, mainly minor bleeding. Dysphagia score improvement was notable, with a standardized mean difference of 1.59, showcasing clinical efficacy [20]. While the MINORS scale indicated moderate to high study quality, the diversity in techniques and potential publication bias necessitate cautious interpretation of the overall positive outcomes [21].

Zenker's diverticulum is a rare esophageal disorder that primarily affects the elderly and is the most common mucosal and submucosal outpouching of the upper gastrointestinal tract [22]. This disorder was first documented in the 18th century, and since then, its treatment and management have evolved [23]. Since 1917, the concept of endoscopic intervention has been presenting encouraging outcomes to the management of ZD, including various non-surgical interventions. The indication for treatment is mainly based on symptomatology and the associated size of the diverticulum. When small (<2 cm) diverticula are incidentally found, no intervention is usually offered; however, the age of the patient and symptoms may prompt a non-invasive intervention, such as local administration of botulinum toxin. Management of larger diverticula usually indicates surgical intervention, as increasing age may be followed by various risk factors that can lead to adverse events due to high risk [24]. More invasive techniques, such as open surgery, usually lead to a more extended hospitalization as well as other common complications of the procedure, such as injury to the laryngeal nerve, esophageal perforation, and extensive bleeding [25,26]. Endoscopic treatment can be superior in high-risk elderly patients who can undergo a brief procedure without general anesthesia in an inpatient or outpatient setting and aggressive manipulation of the cervical spine [27], as recent data have been showing encouraging outcomes of endoscopic intervention, various endoscopic techniques have been suggested, including myomectomy using endoscopic carbon dioxide laser, Harmonic scalpel, needle-knife, hook-knife, monopolar forceps, argon plasma coagulation, as well as an insulated scissor style knife, the SB knife [28].

The complication rate post-treatment was found to be <10% in the cases. Common complications included mostly minor bleeding, micro-perforation, odynophagia, and fever. These findings confirm the existing understanding that myomectomy with an SB knife in ZD can be a safe and effective approach in cases that qualify for endoscopic intervention [29]. Our subjects were mostly individuals >70 years old, with a nearly balanced biological male-to-female ratio: males=60% and females=40%. The above findings were supported by analyzing the reported improvement in symptoms, as reflected in the dysphagia score and the total number of sessions required for management. Though these data were not compared to other endoscopic techniques, our findings produced statistically significant evidence of an efficient myomectomy technique by SB knife in elderly individuals with ZD.

Assessing differences in clinical outcomes, recurrence rates, cost-effectiveness, and complication rates across different endoscopic techniques would surely provide greater insight into how SB knife procedures compare with other existing endoscopic procedures in ZD. Compared to the needle-knife technique, our study shows that the SB knife has comparable clinical success (86% vs. 88%) and recurrence rate (15% vs. 14%), but it is associated with a lower perforation risk (2% vs. 4-6%) [2]. Argon plasma coagulation has been associated with higher recurrence rates (20-25%) than the SB knife (15%) in long-term follow-up studies, suggesting that mechanical septal division may provide more durable results than thermal ablation [30]. Compared with rigid transoral approaches, flexible endoscopic approaches with SB knife avoid general anesthesia and neck hyperextension, making them particularly suitable for elderly patients with comorbidities [8]. Procedure-related morbidity and hospital stay are substantially lower than with surgical approaches [7]. Zenker's peroral endoscopic myotomy (Z-POEM) has also gained widespread popularity; however, head-to-head comparative analysis with the SB knife is limited. A recent comparative study that compared needle-knife septotomy (flexible endoscopic needle-knife septotomy {FENKS}) with Z-POEM found that Z-POEM appears safer than FENKS with similar clinical success rates. Moreover, FENKS was associated with higher adverse events (29% vs. 5%, p=0.018) and hospitalizations (71% vs. 33%, p=0.007) [31]. Although promising, the study had a small sample size. These results underscore the necessity for comprehensive, prospective, and multisite investigations to validate the effectiveness of emerging technologies in ZD treatment and to pinpoint potential recurrence determinants. Meta-regression analyses exploring success and safety revealed that factors such as the cutting device and diverticulum size did not influence the observed outcomes, nor did trial sample size or the anesthesia approach [32].

The strengths of this review lie in its meticulous literature search, clearly outlined inclusion and exclusion criteria, and the meticulous exclusion of redundant studies. Particularly noteworthy is the exclusion of papers utilizing non-rigid devices like harmonic scalpels, preserving a key technique advantage, eliminating the need for neck hyperextension. Additionally, the review boasts a rigorous analysis of study quality. However, the limitations are notable, including a relatively limited number of studies, often constrained sample sizes, the presence of retrospective series, and heterogeneity issues impacting generalizability. Variations in success definitions across studies and the potential for marginal publication bias further contribute to the review's limitations.

Incorporating data from eight studies with 299 patients (60.5% male), the meta-analysis yielded robust outcomes. Procedures lasting 23.06±10.00 min achieved clinical success in 86% after an average of 10.98 sessions, with 22.74% requiring multiple sessions (up to four). The recurrence rate stood at 15%, and intraprocedural complications occurred in 9%, predominantly minor bleeding (9.6%), micro-perforation (2%), odynophagia (1.3%), and fever (1.05%). Late-onset bleeding occurred in 3.2% after one week. Adverse events were conservatively managed. Subgroup analysis by sample size revealed a significant difference in mean sessions (p=0.02). Dysphagia score improvement was notable, with a standardized mean difference (SMD) of -1.59, observed over a mean follow-up of 22.23±11.47 months.

## Conclusions

In conclusion, this meta-analysis strongly underscores the efficacy of SB knife treatment for Zenker's diverticulum, showcasing not only excellent safety but also a significant improvement in dysphagia. While affirming its effectiveness, the study highlights the need for further research to define optimal patient cohorts and to compare its efficacy with that of alternative management techniques.

The discernible heterogeneity observed in our systematic review is predominantly associated with the limited sample sizes in the selected studies. While conducting a randomized controlled trial (RCT) comparing the SB knife treatment to procedures without surgical incisions may raise ethical considerations, there is an imperative need for extensive, forward-looking, and collaborative studies to substantiate the ongoing advancements in SB knife treatment. Moreover, it is essential to augment standardization, particularly in terms of symptom assessment, objective scoring, and precise definitions of achievement, recurrence, and adverse events.
